# Chicago Medical Society

**Published:** 1884-05

**Authors:** 


CHICAGO MEDICAL SOCIETY.

At the regular semi-monthly meeting of March 3, 1884, the large attendance was noted, and the session was unusually interesting. Two important topics were discussed, the first being a rhetorical treatise prepared by Dr. Charles E. Webster, on "Pott's Disease as Illustrating the Principles of Early Diagnosis," containing so much original study that instead of appending a synopsis in these proceedings, the readers of the JOURNAL were favored with the paper in its original form in the April number.

In the discussion which followed a number participated, among whom were Dr. Wm. E. Clarke, who expressed gratification at hearing the paper, and thought the many learned points therein contained should be of much value to the profession generally.

Dr. D. W. Graham had considerable experience in treating this class of troubles. The greater number of his cases had come to him through the dispensary, and were charity patients.

---

April 10. The opacities in the vitreous have entirely disappeared, the fundus is much clearer and vision has decidedly improved.

The speaker thought too much importance could not be attached to early diagnosis in these unfortunate cases, and it should be regarded as a crime for parents to neglect their children at an early stage, or fail to have them treated. The table prepared by the author is admirably gotten up.

Dr. Robert Tilley desired to add his testimony to the value of thepaper,trusting that we should be fully conscientious, especially in this class of cases, to make an early diagnosis. He thought, however, that parents of charity patients did not pay proper heed to advice regarding the welfare of their children.

Two additional members who spoke were at present treating cases of caries in upper dorsal and other regions, that possibly would require the greatest skill in deciding positively whether the disease would invade tissues adjacent,or be retarded,and deformity prevented.

Dr. Webster thought possibly, in conclusion, that muscular rheumatism may be mistaken for Pott's disease, rather than all of those in the table.

This was succeeded by a paper read by Dr. Oscar J. Price, who gave a report of a case of aneurismal tumor from the arch of the aorta, with the causes, varieties, difficulty in diagnosis, treatment, history, and pathology of his case, of which the following main points cited in the paper, with the discussion, are given :

G. R., age 40 ; weight 140 ; temperament, gouty or sanguine; married ; stone contracter ; was first visited by the writer on the morning of February 4 last. The patient had just returned from a Southern trip of some three weeks' duration, having arrived home about two hours previously. Found him sitting up and dressed, quite free from excitement, and apparently in no way entertaining serious apprehension as to his condition or welfare. He had complained more or less during the past year, and there had been constantly a dull aching pain in the muscles of the right arm, which gradually extended to the upper portion of the right chest and centered there. It had become lately quite acute and hard to bear. He also had some cough and dyspnœa ; was conscious of an unusually rapid action of the heart,which particularly attracted his attention upon any exertion or excitement. He stated that his trouble had been considered by his former medical attendant as muscular rheumatism, for which was pursued constant treatment covering many months. Finally he was advised to make a southern trip with hopes of recovering his health. Could not say whether this advice had been given with the belief that there existed a tendency to phthisis. No benefit having accrued from the change of climate as he was conscious of feeling worse, and became more easily exhausted upon the slightest endeavor, he resolved to return home much sooner than he at first contemplated, and while in a sleeping car was suddenly taken with a hæmorrhage from the lungs which was very profuse and exhausting. Receiving some temporary medical attendance at the time he soon rallied from the shock and was enabled to pursue his journey home. In response to inquiries he stated he had generally considered himself rugged and healthy, had led a fairly temperate life. Some twelve years ago, however, he had an attack of acute inflammatory rheumatism, and had also in earlier years contracted syphilis.

A careful and exact examination of his chest was fraught with considerable difficulty, owing to the fact of his having just emerged from a severe attack of hæmorrhage, and the consequent danger of exciting another. Much percussing of the chest not appearing to be warrantable under the circumstances, I was content with as gentle an examination as possible. There was a complete suppression of pectoral or vocal fremitus, and, indeed, a total absence of all sounds over the right thorax as elicited by auscultation. Very gentle and limited percussion appeared to reveal the existence of a cavity of presumably large proportions. In the upper portion of the lung were evidences of broncho-pneumonia, while the heart was found to be crowded considerably to the left; its valves working normally, but much accelerated in action. Instructions were given for the patient to immediately take to his bed, to remain perfectly quiet, and to be given cold drinks and food with a view to avoiding, in so far as possible, other hæmorrhages presumably imminent. In furtherance of this he was placed upon a mixture containing digitalis, ergot, sulphuric acid, and opium, in proper doses, with a view of controlling the excitability and force of the heart's action, allaying pain, and perhaps influencing favorably the expected hemorrhage. The next morning was called early, and found patient had just had another hæmorrhage of about one pint, and was in a state of the greatest prostration, bathed in cold, clammy perspiration, and suffering acute pain, extending from about the second right intercostal space, near the sternum, through to the back. One gr. of ext. ergotine was immediately injected into his back, and ¼ gr. morphia sulph. in his arm. Stimulants were exhibited, and in the course of two hours his condition had materially improved, circulation and warmth being again restored, he resting quite easily. * * * * The following day Dr. J. P. Ross saw the case in consultation, and then a very careful and extensive examination was made. It was his opinion there was an effusion in the right chest, and acting upon this belief, an aspirating needle was thrust through an intercostal space twice at different points, but without return of fluid other than a little blood. No resistance was met by the needle, which after introduction was tipped in different directions without obstruction, thus irresistibly suggesting the conclusion of the existence of a ruptured, leaking aneurism.

Remedies to control the excitability of the heart' action, to allay pain, conjoined with perfect quietude, were all that was now attempted. The following night he had another severe hæmorrhage, from which he only partially rallied, to be plunged into yet another the succeeding day, from which he gradually sank, dying on the evening of the next, a little over a week from the first hemorrhage.

Autopsy eighteen hours after death kindly permitted by friends. Besides the author, there were present Drs. J. P. Ross, J. A. Robison, and E. P. Murdock; Rigor mortis well established; subject fairly nourished; skin white. Incision from upper part of sternum to ensiform cartilage, thence along course of costal cartilages. Incision into right pleural cavity caused a small amount of sero-sanguineous fluid to flow out; heart revealed crowded to the left, and large blood clots in right pleural cavity; blood removed to the amount of over two quarts. There now appeared a large sacculated aneurism of the first portion of the arch of the aorta, enclosing a large fibrinous mass some four inches in diameter. Pericardium adherent to heart; sac of aneurism adherent to right pleura; post-mortem clot in right auricle; aneurism ruptured, opening into the pleural cavity at the lower portion of upper lobe of right lung in front, also an opening into the upper portion of the right upper lobe communicating with a bronchial tube, the lung being filled with blood, completely collapsed, and its tissue very fragile. The heart was enlarged and dilated, the walls of the left ventricle being thin and infiltrated with fat, especially toward apex. Very slight fibrous deposit, or vegetation, in one aortic valve. The sac of aneurism so large as to contain a man's double fist, its distended, its vascular coverings having undergone such changes as to render them quite unrecognizable. The inner aspect presented a rough, uneven surface, composed of laminated fibrin and fresh blood clot. These layers of fibrin had been built upon each other into liberal proportions, so as to virtually constitute all that remained of the aneurismal sac.

(The specimen was then presented for observation.) In contemplating this case, it is interesting to observe the length of time the patient survived after such a formidable rupture, which evidently ushered in the first hæmorrhage, the inference being that the heart, temporarily relieved of its force and impetus, permitted a large-sized coagulum to block up the opening for the time, subsequent hæmorrhages resulting from the instability of the same, which doubtless was considerably guarded against by perfect repose and quiet. The usual connection between aortic aneurism and ventricular hypertrophy is again illustrated in this case. There had also been, as already stated, pericarditis, which presumably took its origin from the severe attack of inflammatory rheumatism several years before, although it is perhaps questionable if the propulsive action of the heart alone is able to produce aneurism, unless of a diseased artery. It is reasonable to suppose, however, that a disease which may produce endocarditis and pericarditis may also cause endoarteritis. The history of the case also furnishes another somewhat commonly accepted cause for aneurism, negative testimony to the contrary. Military statistics would appear to demonstrate that a large number of cases of inflammatory involvement of blood-vessels, ending in atheroma or fibrous thickening of the coats of the vessels are of syphilitic origin. Admitting that this is by no means established, it would still appear that it is a disease, in common with rheumatism, tending to impair not only the functional properties, but the nutrition and structure of arterial walls, and is thus justly entitled to due consideration as a possible element in the case. Reasoning by exclusion, the patient had not been addicted to more than ordinary, or a very moderate indulgence in alcoholic drinks, and, therefore, can hardly be said to have furnished this supposedly frequent factor to the malady. Muscular effort or over-exertion also, as superinducing elements, appear to have no proper claim to consideration, as the patient had for fifteen years preceding death led a very quiet life, relieved from manual labor. In estimating the general causes of aneurism, it is of course an open field for conjecture, and capable of many diverse illustrations. Still, it would appear that the history of this case furnishes one, if not two, very reasonable and commonly accepted conditions leading to its development. Diseases which frequently erminate in a chronic inflammation of the internal coat of an artery may be properly classed as predisposing to aneurism, and such this case affords. Aside from traumatic origin, it is evident that structural lesions of a destructive tendency, involving the middle and internal coats of arteries, play an important part in the production of aneurism.

Thus we have the conditions most favorable to the formation of plastic deposits on the free surface, which are so commonly met with in the aorta or large vessels. From this plastic inflammatory process, commencing in the lining membrane, morbid changes are initiated involving the middle coat, which, becoming thinned by pressure, soon softens and yields, forming a kind of pouch or depression. Firm adhesions are now found between the two membranes, and we have the commencement of an aneurism. It is evident the natural tendency of such a destructive process would be to rupture, but in accordance with the wonderful preservative powers of nature, conservative forces are set into action, also the external coat becomes thickened, indurated, and supported by this deposition of plastic matter going on beneath it, until it becomes a firm and rugged barrier thus preventing for a long time, and sometimes permanently, the formation of the sac. It is, however, inelastic, and performs no proper part in the distributing of blood which passes through it. Its caliber, therefore, must gradually dilate, its pouch-like form becoming the natural receptacle for the deposition of coagula and fibrous deposit, and the specimen affords an excellent observation of the laminated type built upon itself layer upon layer, adhering firmly to the interior ot the sac, and arranged concentrically like the coverings of an onion. Its large size would seeme to indicate it had been a long time in forming.

DISCUSSION.

Dr. R. Tilley thought if syphilis was a cause of aneurism (which was remote in this case, the disease having existed prior to 12 years ago), it did not establish the fact that the man had an aneurism.

Dr. H. J. Reynolds asked if there were no murmurs present? Answered, no. There were no sounds in the right portion of the thorax. The heart was crowded out of its place (to the left) and enlarged.

Dr. P. C. Jensen inquired of the author if there were ever present girdled pains around the patient's chest at night? Answered, would hardly regard them as such. There was a good deal of pain in the right chest in the second intercostal space, especially when the violent hæmorrhages occurred.

Dr. W. L. Axford thought aneurism was not always easy to diagnosticate. The great Liston once opened what he supposed to be a boil on the chest of a man, when the patient at once sank, bleeding to death, caused by an aneurism that had been cut into.

Dr. C. E. Webster asked if there was any erosion of any of the vertebræ, or symptoms of erosion of the sternum present?

Answered no, not the least.

Dr. W. E. Clarke asked, how long before death was the patient aspirated? Answered, 36 hours.

Dr. L. H. Montgomery asked if the patient had taken iodide of potassium for rheumatism, and for the specific disease, as well probably as for the influence it may exert over aneurisms, and further, was there no incipient stage of the aneurism present a year ago?

Regarding the case reported, I am well acquainted with a brother of the deceased; a gentleman who is a carrier in the post-office, and whose family I have attended for several years. He told me that nearly two years ago the deceased had witnessed a sad accident in which his 12-year-old son had been killed; that his body was mangled horribly while he stood but a few feet away at the time the accident occurred, and he saw his boy's head crushed off. The father stood apparently motionless and speechless, his features becoming very pale at the ghastly sight he beheld. If this be so, could not the heart's action temporarily intermit, or cease to beat for a few pulsations, caused by a sort of paralysis, the result of what he had seen? It was from about this date that he had complained of more or less pain in the side and chest.

Dr. Price, in closing, stated, in reply to the last speaker, that he had treated the patient a year ago, and gave him iodide of potash in 10-grain doses three or four times a day for four weeks. Secondly, he thought the accident which had just been spoken of might, possibly, have produced the starting or incipient stage of the aneurism; and, thirdly, while the deceased was South, some ten days before death, he engaged in violent exercise, that of jumping 8 feet 2 inches. This he did twice, and became ill shortly after.

Upon motion the society adjourned.

L. H. M.

---

THE CHINESE METHOD OF DETERMINING PATERNITY.—A correspondent (J. H. Lowry), of the *Lancet*, gives the following bit of medico-legal evidence: A basin or cup of clean water is obtained; the supposed father's finger is cut and then put into the water till some blood trickles; then the child's finger is cut and placed in the water, and if the two bloods immediately unite the proof is complete. The magistrate is sometimes bribed and the water tampered with.

